# Targeted re-sequencing of *F8*, *F9* and *VWF*: Characterization of Ion Torrent data and clinical implications for mutation screening

**DOI:** 10.1371/journal.pone.0216179

**Published:** 2019-04-26

**Authors:** Eric Manderstedt, Rosanna Nilsson, Christina Lind-Halldén, Rolf Ljung, Jan Astermark, Christer Halldén

**Affiliations:** 1 Department of Environmental Science and Bioscience, Kristianstad University, Kristianstad, Sweden; 2 Department of Clinical Sciences–Pediatrics and Malmö Center for Thrombosis and Hemostasis, Skåne University Hospital, Malmö, Sweden; 3 Department for Hematology Oncology and Radiation Physics, Center for Thrombosis and Hemostasis, Skåne University Hospital, Malmö, Sweden; Institut d'Investigacions Biomediques de Barcelona, SPAIN

## Abstract

Mutations are not identified in ~5% of hemophilia A and 10–35% of type 1 VWD patients. The bleeding tendency also varies among patients carrying the same causative mutation, potentially indicating variants in additional genes modifying the phenotype that cannot be identified by routine single-gene analysis. The *F8*, *F9* and *VWF* genes were analyzed in parallel using an AmpliSeq strategy and Ion Torrent sequencing. Targeting all exonic positions showed an average read depth of >2000X and coverage close to 100% in 24 male patients with known disease-causing mutations. Discrimination between reference alleles and alternative/indel alleles was adequate at a 25% frequency threshold. In *F8*, *F9* and *VWF* there was an absolute majority of all reference alleles at allele frequencies >95% and the average alternative allele and indel frequencies never reached above 10% and 15%, respectively. In *VWF*, 4–5 regions showed lower reference allele frequencies; in two regions covered by the pseudogene close to the 25% cut-off for reference alleles. All known mutations, including indels, gross deletions and substitutions, were identified. Additional *VWF* variants were identified in three hemophilia patients. The presence of additional mutations in 2 out of 16 (12%) randomly selected hemophilia patients indicates a potential mutational contribution that may affect the disease phenotype and counseling in these patients. Parallel identification of disease-causing mutations in all three genes not only confirms the deficiency, but differentiates phenotypic overlaps and allows for correct genetic counseling.

## Introduction

Inherited bleeding disorders affect an estimated 7.5 million individuals worldwide [1]. The common inherited bleeding disorders, von Willebrand disease (VWD), hemophilia A (HA) and hemophilia B (HB), account for 95–97% of such patients [2]. VWD is the most common inherited autosomal bleeding disorder, affecting both genders. Mutations in the *VWF* gene result in quantitative or qualitative deficiencies of von Willebrand factor (VWF). The estimated prevalence of subjects with significant bleeding symptoms is 1 in 1000 [3]. More than 700 mutations have been associated with VWD (https://grenada.lumc.nl/LOVD2/VWF/home.php?select_db=VWF). HA and HB are X-linked recessive disorders caused by mutations in the *F8* and *F9* genes, resulting in quantitative or qualitative defects in factor VIII (FVIII) and factor IX (FIX), respectively. The prevalence of the disorders is 1 in 5000 (HA) and 1 in 30,000 (HB) male births [1]. A recurring inversion located at intron 22 in *F8* accounts for approximately 45% of all severe HA cases. Additionally, more than 2000 point mutations and indels distributed throughout the gene have been associated with the majority of the remaining HA cases (http://www.factorviii-db.org). More than 1000 point mutations and indels have been reported in *F9* in patients with HB (http://www.factorix.org).

Pathogenic variants are identified in approximately 95% of HA cases and in almost all of patients with HB. Misdiagnosis due to overlapping phenotypes as well as deep intronic variants altering mRNA splice sites may account for the remaining mutation-negative cases [1]. In 10–35% of the type 1 VWD index cases, no pathogenic variant can be identified. In the majority of these cases, a mild bleeding phenotype is observed making diagnosis a challenge [4]. However, a more stringent definition of type 1 VWD patients with VWF level ≤30 IU/dL identified mutations in ~92% of patients [5]. In addition, intronic variants as well as variants at other genetic loci may affect the VWF level. For example, in a meta-analysis of several genome-wide association studies, eight genes that contribute to plasma level variation of VWF were identified [6]. A common variant in *ABO* showed the strongest effect, but smaller effects were seen for common variants in *CLEC4M*, *SCARA5*, *VWF*, *STAB2*, *STX2*, *TC2N* and *STXBP5*.

Additional difficulties arise from distinguishing genocopy disorders such as type 2B VWD and PT-VWD where the clinical picture is similar but results from different genetic causes [1]. Another example is type 2N VWD and mild HA which both present with low FVIII activity [7]. The bleeding tendency also varies among patients carrying the same causative mutation, potentially indicating additional genes modifying the phenotype which cannot be identified by routine single-gene analysis [6]. In all three disorders, genetic testing provides confirmation of deficiency, differentiation of overlapping phenotypes, as well as genetic counseling.

Historically, the first line of approach for diagnosing inherited bleeding disorders has been based on phenotypic assays assisted by sequencing of the phenotypically indicated gene for confirmation [1]. Recently a number of next generation sequencing (NGS)-based studies have been used to re-sequence panels of genes associated with inherited bleeding disorders in a single workflow. Bastida et al. [8] designed a gene panel targeting *F8*, *F9* and *VWF* on an Illumina MiSeq platform whereas Bastida et al. [9] used a 23-gene panel to target both common and rare bleeding disorders using the same platform. The ThromboGenomics initiative targeted 63 genes associated with inherited bleeding, thrombotic and platelet disorders [10].

Ion Torrent is an NGS platform based on semiconductor chip technology [11, 12]. Using the AmpliSeq strategy, targeted re-sequencing is performed through the simultaneous amplification of many amplicons in a multiplex PCR. Adapters and barcodes are ligated to the fragments which are subsequently enriched using a few PCR cycles. The library of adapter-containing fragments is used to populate spheres through emulsion PCR. These PCR steps result in incorporation of errors in varying proportions of the reads.

To enable simultaneous mutation detection in the three common inherited bleeding disorders, the present study re-sequenced the exonic positions of *F8*, *F9* and *VWF* using Ion Torrent sequencing based on an AmpliSeq strategy and made a quality assessment of the data obtained. To further validate the sequencing system a total of 24 subjects previously diagnosed with HA, HB or VWD were analyzed for their known mutations.

## Materials and methods

### Study populations

The retrospective VWD study population was recruited at the Department for Coagulation Disorders, Malmö University Hospital (Malmö, Sweden). Clinical and laboratory data were recorded and bleeding phenotypes were classified [13]. The retrospective HA and HB populations were recruited at Malmö Hemophilia Centre at their routine visit and characterized with respect to their mutations [14, 15]. Eight randomly selected patients were selected from each of the VWD, HA and HB populations described above and included in the present study. The study was approved by the Regional Ethical Review Board in Lund under Dnr LU 2015/820, 2017/278 and 436–01. Written informed consent was obtained from all participants.

### Ion Torrent sequencing

The primer sets were designed using Ion AmpliSeq Designer to include all exonic, 5’UTR and flanking intronic sequences (http://www.ampliseq.com, pipeline version 2.2.1). The multiplex primer pools were optimized such that the primers systems with the lowest read depths in a specific pool were added to the other pool, excluding overlapping systems. The library preparation was achieved using the Ion AmpliSeq library kit 2.0 according to the manufacturer’s protocol (Life technologies, Carlsbad, CA, USA). The amplicons were barcoded using Ion Xpress barcode adapters. Purification of the library was achieved using Agentcourt AMPure XP reagent beads (Beckman Coulter Inc., Brea, CA, USA). Library amplification was performed and further purification steps were achieved using Agentcourt AMPure XP reagent beads before elution of the final library. The library concentrations were determined by capillary electrophoresis using a Fragment Analyzer (Advanced Analytical Technologies, Ankeny, IA, USA) and a High Sensitivity NGS Fragment Analysis kit (Advanced Analytical Technologies). Library normalization was performed to a concentration of ~50 pM before being pooled together. An amplification reaction was prepared according to the manufacturer’s protocol and transferred to an Ion PGM Hi-Q View reaction filter before emulsion PCR was performed on an Ion OneTouch 2 instrument (Life Technologies). Enrichment of the Ion Sphere Particles (ISPs) was performed on an Ion OneTouch ES instrument (Life Technologies) using Dynabeads MyOne Streptavidin C1 beads. The sequencing process was carried out on an Ion PGM sequencer (Life Technologies) using Ion 316 chip V2 (Life Technologies). This allowed simultaneous analysis of eight samples at the coverage presented in this study. The loaded chip was sequenced using a 400 bp sequencing protocol with 850 flows of single nucleotides.

### Bioinformatic analysis

The generated raw data was processed by the Ion Torrent Suite Software v5.0.5. The sequences were aligned to the Homo sapiens hg19 reference genome and stored as BAM files. The frequency parameter for variant calling was set to 0.25, generating VCF files for each library containing SNPs, small insertions and deletions (indels) and putative mutations. The generated VCF files were merged and multi-allelic sites split to create a database containing all called variants. Annotation of the database VCF file was achieved through the Variant Effect Predictor, VEP [16]. Evaluation of detected variants of each library was performed in parallel using MuCor: Mutation Aggregation and Correlation [17]. Initially, a setup script was run, generating a JSON configuration file containing library IDs and associated VCF and BAM files as well as a link to the VCF database. Secondly, the run script was implemented, analyzing the reported variants using the JSON file. The generated output files contained variant reports, location and annotation for variants of each library (S1 Table). The non-Finish European (NFE) population of the Exome Aggregation Consortium database, ExAC[18], was used to filter the initial set of variants to identify possible disease-causing variants. This was achieved by eliminating all variants with an allele frequency of >1%. A BED file with the exonic positions of *F8*, *F9* and *VWF* was obtained from UCSC genome browser [19]. Mpileup files were generated using the SAMtools mpileup application, BAM files from each library and the BED file with the exonic positions. Base calls for each DNA strand, reference base, read depth and position were extracted from the mpileup format for further analysis (S2 Table, S3 Table and S4 Table).

### Data analysis

Evaluation of the gene panel was achieved by using the generated data from the 24 subjects. All the calculations were performed on an individual level as well as for a system average using RStudio [20]. Basic descriptive statistics of the panel included read depth (the number of times each base position was interrogated), read depth variation, coverage (the number of base positions interrogated out of all attempted), strand bias (the relative number of reads for each strand), reference allele (true alleles and polymorphisms) frequencies and alternative allele/indel frequencies. The alternative alleles may originate from errors occurring during PCR amplification or be the result of existing mosaic variants. The average read depth for each sample was calculated and used to normalize the read depth of each position. The variation of the read depth was calculated by normalization of each sample library. The coverage was calculated by dividing the number of bases fulfilling the criteria with the total number of bases. A strand bias ratio was calculated by dividing the number of read bases of the forward strand by the total number of read bases in each position. The obtained percentage represented the forward strand, while the complement represented the reverse strand. The alternative allele frequency was determined by recovering the called bases, which differed from the nucleotide with the highest number of calls. By dividing the number of alternative alleles by the total number of alleles, the frequency as well as the standard deviation could be determined for each position. If the calculated alternative allele frequency was >0.25, a recalculation was made using the two alleles with the lowest number of calls. The indel frequency for each position was calculated by using the same strategy. Mutations and indels associated with HA, HB and VWD were obtained from the variant databases and were evaluated using the calculated alternative allele/indel frequencies. The allele frequency for *F8* and *F9* was determined by obtaining the nucleotide with the highest number of calls for each position and dividing by the total number for each position. The minor allele frequency for *VWF* in a heterozygote was determined by obtaining the nucleotide with the second highest number of calls and dividing by the total number for each position. Positions with MAF <0.25 were eliminated from the calculation.

## Results

### Read depth and coverage

*F8*, *F9* and *VWF* were analyzed in a single workflow using an AmpliSeq panel targeting the exons and flanking intronic regions of the three genes. Data were obtained from 24 male patients with known disease-causing variants. The panel targeting all the exonic positions showed an average read depth of 2122X. Since *VWF* is located on chromosome 12, twice as many reads were obtained compared to the X-chromosome genes, *F8* and *F9*. The coverage over *F8*, *F9* and *VWF* was close to 100% in all three genes. The missing bases were due to low yielding primer systems excluding eight nucleotides located in the 5’ UTR region of *F8* and the 216 bp of exon 15 in *VWF*. Given an average read depth of ~2000X, only exon 15 of *VWF* showed an average read depth of <100X. Both between-individual and between-amplicon variations were fairly large but unproblematic, due to the high average read depth (S1A Fig). Only a few positions showed a strand bias of >95% (S1B Fig). The results for *F9* were similar to the results of *F8* (results not shown).

### Reference and alternative alleles

**Fig 1:**
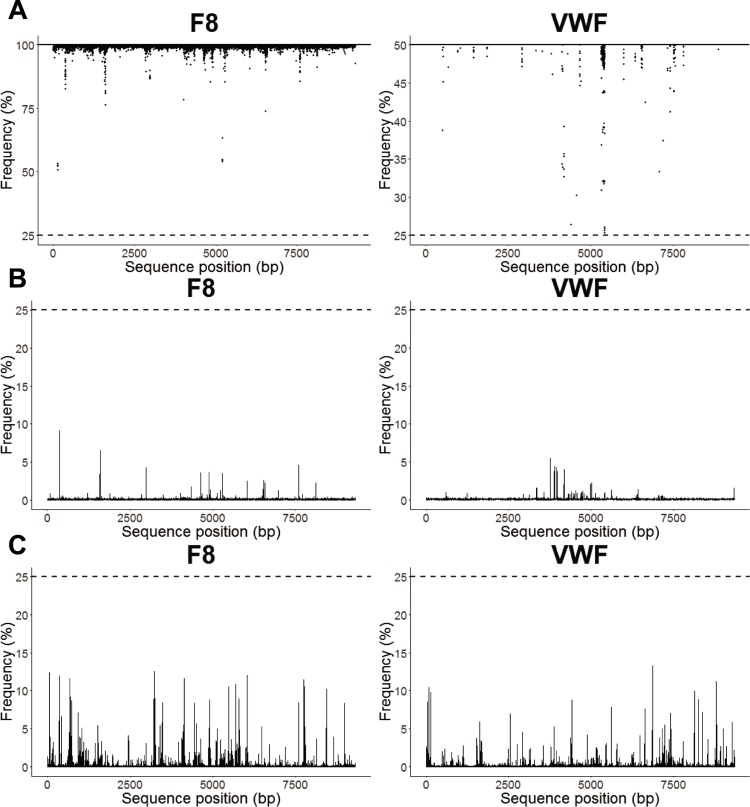
Reference allele and alternative allele/indel frequencies observed for all positions of *F8* and *VWF* for all subjects. (A) Reference allele frequencies. Expected frequencies for hemizygote (*F8*) and heterozygote (*VWF*) positions given by solid black line. (B) Average alternative allele frequencies. (C) Average indel frequencies. Noise level limit at 25% given by dashed line.

Discrimination between reference alleles and alternative alleles/indels was adequate at a 25% frequency level (Fig 1). In *F8* an absolute majority of all reference alleles was present at allele frequencies >95%. Only at ~10 positions did a few individuals show allele frequencies of <95% (Fig 1A). No position showed a reference allele frequency <50% for any individual. In *F8* the average alternative allele and indel frequencies never reached above 10% and 15%, respectively (Fig 1B and 1C). Less than 0.1% of the total data set (all combinations of positions times individuals) showed alternative allele and indel frequencies in the range of 10–25% when individual patients were evaluated. *F9* showed a similar result (results not shown). This provided confident discrimination between reference and alternative alleles in an absolute majority of cases.

In *VWF*, all homozygote positions showed a similar pattern as seen in *F8* and *F9*. The minor reference allele frequencies are shown as dots in Fig 1A for each individual who was heterozygous for a given position. Also in this case, few reference allele frequencies decreased more than 5%, except for 4–5 regions which showed lower frequencies; in two cases they were even close to the 25% cut-off for reference alleles. These were sequences within the pseudogene region where the necessary filtering complicates allele calling. For *VWF* the average alternative allele and indel frequencies were well below 10% and 15%, respectively (Fig 1B and 1C). Thus, overall there were only a very few positions in *F8*, *F9* and *VWF* that it was difficult to call reference alleles for. Comparison of the alternative allele and indel frequencies indicated that indel biases were occurring more frequently and showed a greater variation (Fig 1B and 1C).

To further investigate the positions that are relevant in a mutation screening perspective, all alternative allele/indel frequencies for the disease-causing positions in the mutation databases (http://www.factorviii-db.org, http://www.factorix.org and https://grenada.lumc.nl/LOVD2/VWF/home.php?select_db=VWF) were collected. The absolute counts and frequencies of alternative allele/indel frequencies are given in Table 1 for all mutated positions.

**Table 1:** Alternative allele/indel frequencies in mutated positions collected from gene-specific databases, http://www.factorviii-db.org, http://www.factorix.org and https://grenada.lumc.nl/LOVD2/VWF/home.php?select_db=VWF.

Alternative	Point mutation		Indel	
frequencies (%)	Absolute	Frequency (%)	Absolute	Frequency (%)
**<1**	2029	99	533	96
**>1**	12	0.58	20	3.6
**>2**	8	0.39	9	1.6
**>5**	1	0.05	4	0.72
**>10**	0	0	2	0.36

Overall, the majority of mutations are located at positions with a low alternative allele frequency of <1% and only a few remain at higher frequencies. The patterns of alternative allele and indel frequencies largely resembled the corresponding patterns for all gene positions visualized in Fig 1B and 1C. Thus, the mutated positions in the mutation databases were not more challenging than the average position with regard to allele calling and discrimination of alternative alleles and indels.

### Detection of mutations

The random selection of 24 patients each presenting with either HA, HB or VWD had mostly substitution mutations, but three small indels and three gross deletions were also represented. In different patients, a heterozygote deletion of exon 14–52 in *VWF* (patient VWF_25) and hemizygote deletions of the entire *F9* (HB_129) and exon 1–12 of *F8* (HA_420) were all detected by a decreased or absent read depth of all involved amplicons. The remaining 21 patients all presented with different substitution and indel mutations. All but one of these variants were identified immediately by annotation through VEP. In one case (HA_208) the known mutation could be detected only after careful inspection of the data. The disease-causing variant was located at a homo-polymeric region, consisting of a 9-nucleotide long poly-T stretch. In this case the relative flow value change for consecutive alleles decreased to an extent where it became a challenge to determine the correct number of incorporated nucleotides. Table 2 provides a summary of the known and detected mutations together with read depths and strand biases for all mutations. The strand biases clustered around the expected value of 0.5, with only one value (0.35) deviating more than 10% from the expected value. The read depths were all fairly high, ranging from 80X to >5000X. Thus, at an average read depth of ~2000X none of the allele calls were problematic.

**Table 2:** Detected disease causing substitutions, indels and large deletions in the 24 patients.

Patient ID	Gene	Detected/known mutation	Protein	Consequence	Phenotype^a^	Quality^b^
HA_208	*F8*	c.3637dupA	p.Ile1213AsnfsTer28	Frameshift	Mild-Severe (32)	283/0.52
HA_365	*F8*	c.3640del	p.Gln1214ArgfsTer4	Frameshift	-	454/0.45
HA_398	*F8*	c.67A>G	p.Arg23Gly	Missense	Mild (2)	3764/0.57
HA_408	*F8*	c.5381T>A	p.Phe1794Tyr	Missense	-	2830/0.43
HA_420	*F8*	Gross deletion	-	Gross deletion	Severe (-)	0/0
HA_432	*F8*	c.923C>T	p.Ser308Leu	Missense	Severe (28)	3660/0.56
HA_448	*F8*	c.3134delC	p.Pro1045HisfsTer8	Frameshift	-	581/0.42
HA_459	*F8*	c.5393C>T	p.Ala1798Val	Missense	Moderate (1)	2416/0.42
**HA_459**	***VWF***	**c.5453A>G**	**p.Asn1818Ser**	**Missense**	**Type 1 (1)**	**367/0.49**
HB_129	*F9*	Gross deletion	-	Gross deletion	Severe (60)	0/0
HB_130	*F9*	c.572G>A	p.Arg191His	Missense	Mild-Severe (85)	3098/0.51
HB_132	*F9*	c.82T>C	p.Cys28Arg	Missense	Mild-Moderate (5)	420/0.46
HB_135	*F9*	c.785T>C	p.Ile262Thr	Missense	Mild-Moderate (6)	474/0.58
**HB_135**	***VWF***	**c.8084C>G**	**p.Pro2695Arg**	**Missense**	**Unclassified (1)**	**787/0.5**
HB_136	*F9*	c.459G>A	p.Val153 =	Silent	Mild (6)	2670/0.45
HB_138	*F9*	c.1135C>T	p.Arg379Ter	Nonsense	Mild-Severe (65)	887/0.5
**HB_138**	***VWF***	**c.7940C>T**	**p.Thr2647Met**	**Missense**	**Type 1 (2)**	**3533/0.35**
HB_139	*F9*	c.1289G>T	p.Ser430Ile	Missense	Moderate (1)	415/0.47
HB_140	*F9*	c.83G>A	p.Cys28Tyr	Missense	Moderate-Severe (9)	1575/0.48
VWF_25	*VWF*	Gross deletion	-	Gross deletion	Type 3 (-)	-/-^**c**^
VWF_140	*VWF*	c.5014G>A	p.Gly1672Arg	Missense	Type 2A (1)	5202/0.49
VWF_159	*VWF*	c.4120C>T	p.Arg1374Cys	Missense	Type 1, 2A, 2M (7)	347/0.53
VWF_166	*VWF*	c.7603C>T	p.Arg2535Ter	Nonsense	Type 3 (4)	407/0.45
VWF_172	*VWF*	c.4975C>T	p.Arg1659Ter	Nonsense	Type 3 (10)	1595/0.57
VWF_189	*VWF*	c.4517C>T	p.Ser1506Leu	Missense	Type 2A (14)	4821/0.41
VWF_238	*VWF*	c.2278C>T	p.Arg760Cys	Missense	Type 2N (1)	80/0.53
VWF_280	*VWF*	c.7430G>C	p.Cys2477Ser	Missense	Type 1 (1)	270/0.41

Additional variants marked as bold.

^a^ Phenotype is given as degree of severity for HA and HB, whereas VWD is classified by subtype. Number of reported cases given within parentheses.

^b^ Quality parameters given as read depth and strand bias for the mutated positions.

^c^ Deletion encompassed exon 14–52 and all amplicons involved showed approximately 50% of the average read depth and a similar strand bias compared to the remaining patients.

One HA patient and two HB patients had, in addition to their previously known mutations in *F8* and *F9*, single variants in *VWF*. Patient HB_138 had in addition to a nonsense mutation in *F9* a *VWF* missense mutation (c.7940C>T) reported as a type 1 VWD mutation. Clinical data for this patient showed a severe phenotype (FIX:C, <1%; FIX:Ag, <1%). Patient HB_135 had a missense mutation in *F9* and another missense variant (c.8084C>G) in *VWF*. Clinical data for this patient showed a moderate phenotype (FIX:C, 4%; FIX:Ag, 4%). Lastly, patient HA_459 with a missense mutation in *F8*, also had another missense variant (c.5453A>G) in *VWF* previously reported as a type 1 VWD mutation. Clinical data for this patient showed a mild phenotype (FVIII:C, 30%).

## Discussion

### Read depth and coverage

Targeted re-sequencing of *F8*, *F9* and *VWF* was optimized and validated in the present study using an AmpliSeq-based Ion Torrent sequencing strategy. Two key parameters used in NGS to evaluate the results are read depth and coverage. The read depth can be adjusted through selecting chip size and number of samples sequenced in parallel on each chip. Given a primer design capable of amplifying all targeted regions, the normalization of the different amplicons is key to an efficient utilization of the available sequencing capacity. To achieve this, the multiplex primer pools were optimized so that the primers systems with the lowest read depths in a specific pool were added to the other pool, excluding overlapping systems. Further optimization adjusted the concentrations of individual primer pairs. This resulted in a total coverage over *F8*, *F9* and *VWF* of >99%. The major exception was the 216 bp of exon 15 in *VWF* that was not well covered. This corresponds to a single amplicon with a decreased amplification efficiency. As the gene panel contains 123 amplicons the effect of this shortcoming is fairly limited. The VWD mutation database (https://grenada.lumc.nl/LOVD2/VWF/home.php?select_db=VWF) contains a total of 1437 reported individuals distributed over 708 different mutated positions (1193 database entries; access date 21-03-2019). The mutations in exon 15 represent a small fraction of the total count, with 22 mutations all reported in single cases. However, it is an obvious shortcoming of the technique for which it will need to be corrected. In contrast, the Illumina platform was used by Bastida et al. [8] to perform hybridization-based targeted resequencing of *F8*, *F9* and *VWF*. They determined their coverage over the same target regions to 90.5% with a read depth of 50X, without giving further details of the system characteristics. The studies use different enrichment strategies of the target regions. AmpliSeq uses PCR amplification based on pools of primers whereas the Illumina system of Bastida et al. [8] used hybridization-based enrichment. With the AmpliSeq strategy a higher coverage was achieved (99%), though at a cost of lower uniformity, necessitating a higher average read depth to reliably call variants across individuals [21].

In multiplex PCR-based systems like the AmpliSeq/Ion Torrent system all amplicons compete with each other during the amplification process. This is an inherent property of multiplex PCR and is expected in situations where large numbers of primer pairs are present in the same reaction. The amplification profiles of different samples are similar. This means that the differences observed are to a large extent reproducible and therefore systematic in nature and not the result of spurious variation. Thus, an even read depth for all amplicons is primarily determined by the normalization of all amplicons by the initial pool optimization. Given basal amplicon optimization, the most important parameter influencing read depth within and between different samples is the normalization to equimolar amounts of individual libraries. The obvious ideal is total equimolarity at all stages of pool optimization and normalization. Failure to achieve full coverage can in most cases be compensated for by simply increasing the total number of reads.

### Reference and alternative alleles

The results of the present study showed that discrimination between reference alleles and alternative alleles/indels was clearly sufficient in an absolute majority of cases. However, there were a few positions in single individuals where reference alleles were difficult to distinguish from alternative alleles. These included a small number of mononucleotide repeats of sizes >6–9 repeat units. Such cases required determination of whether the alternative allele frequency reflected an error, or if it was a true alternative allele. In this project the threshold for variant calling was set at 25%. Higher frequencies were further examined to determine if they were a true variant or not. VEP was the first application used during the mutation identification process, where variants were filtered using the ExAC allele frequencies. ExAC allele frequencies have collected sequence data from different global projects and have created a register containing both neutral and polygenic variants. The efficiency of the VEP application eliminated a majority of all variants and left only one or a small number of possible disease-causing mutations for further analysis.

### Detection of mutations

All mutations for *F8*, *F9* and *VWF* were extracted from the locus-specific mutation databases. Since the three databases contain high numbers of mutations they are likely to contain a majority of the disease-causing variants in these genes. The majority of these positions showed an alternative allele frequency of <1% and were therefore not more difficult to analyze than the average position of the whole coding sequence. Furthermore, none of the frequently reoccurring mutations (> 50 reported cases) associated with the common bleeding disorders were located in positions with alternative allele frequencies >1%. Thus, the commonly mutated positions of these genes present with less of a challenge with regard to allelic discrimination than the average position.

As Sanger-based resequencing only sequences the phenotypically indicated gene, it can lead to incorrect diagnosing as well as therapy. Bastida et al. [8], described the importance of differentiation between the VWD-2N and the mild/moderate HA phenotypes. They also demonstrated the value of using gene panels as no mutation was found in *F8* in eight previously diagnosed mild HA cases. In these cases, mutations associated with VWD-2N were identified, resulting in a reclassification. Another recent study by Borras et al. [5] reclassified 110 out of 556 patients after NGS resequencing. In the present study, additional *VWF* variants were detected in three different hemophilia patients. Patient HB_138 had in addition to a nonsense mutation in *F9* (c.1135C>T; previously reported 65 times in *F9* mutation database) a *VWF* missense variant (c.7940C>T) reported as a type 1 VWD mutation in two cases. The phenotypic data for this patient showed a severe phenotype (FIX:C, <1%; FIX:Ag, <1%, [22]) like most of the patients listed in the FIX mutation database for this position (http://www.factorix.org). Patient HB_135 had a missense mutation (c.785C>T) previously reported in six mild-moderate HB patients. Phenotypic data for this patient showed a moderate phenotype (FIX:C, 4%; FIX:Ag, 4%, [23]). In this patient another missense variant (c.8084C>G) was detected in *VWF*. This variant was reported in a single control individual and classified as probably not pathogenic [24]. Finally, patient HA_459 with an *F8* c.5393C>T missense mutation reported in a single patient with moderate disease, also had another variant (c.5453A>G) in *VWF* previously reported as a type 1 VWD mutation. Phenotypic data for this patient showed a mild phenotype (FVIII:C, 30%). The single listing in the FVIII mutation database (http://www.factorviii-db.org) reports moderate severity (FVIII:C, 3%). Thus, no conclusive impact of *VWF* variants could be detected. At present it is unknown if these *VWF* variants are indeed contributing to disease, but two of them have been reported as being type 1 VWD mutations. It is highly likely that both *F9* mutations, c.1135C>T previously reported 65 times and the c.785C>T mutation previously reported six times, are true mutations. The c.5393C>T missense mutation in *F8* reported in only a single patient is more doubtful. Since the three *VWF* variants have all been detected previously in only one or two individuals, their mutation status is also uncertain. However, all three variants had read depths and strand biases allowing an interpretation as true alleles and were in addition validated by Sanger sequencing. The findings encourage further studies to evaluate the presence of additional potentially disease-causing variants that may contribute to the phenotype in such patients. This may also contribute information with regard to the varying bleeding tendency seen among patients carrying the same causative mutation.

Some of the important aspects when dealing with a bleeding disorder are distinguishing genocopies, risk of developing alloantibodies, prenatal diagnosing and identification of carriers. The analyses of these aspects all depend on the same thing: to identify the candidate mutation. Ion Torrent provides a high throughput method suitable for identification of mutations in a clinical setting. The AmpliSeq/Ion Torrent strategy enables multiplex gene analysis and multiplexing of samples which reduces the amount of labor and provides a rapid turnaround time.

## Supporting information

S1 Table Identified variants with all annotation from all individuals. (XLSX)

S2 Table Primary data for HA patients including read depth data for all positions in and reported nucleotides on both strands for all individuals. (XLSB)

S3 Table Primary data for HB patients including read depth data for all positions in and reported nucleotides on both strands for all individuals. (XLSB)

S4 Table Primary data for VWD patients including read depth data for all positions in and reported nucleotides on both strands for all individuals. (XLSB)

S1 Fig Average read depth and strand bias for all positions of all amplicons in *F8* and *VWF*. (A) Average read depth, 100 reads given as a dashed red line. (B) Strand bias, bias exceeding 19:1 in either direction given as a dashed red line.
(EPS)
